# 

**Published:** 1988

**Authors:** 


					)2 VI BR323
^ NO.1a 1983
SEO- B33740000
IsTOL MEDICO-CHISURGICAL
MEDICO
CHIRURGICAL
JOURNAL
SPECIAL SUPPLEMEIMT
v?LUME 102( 1 a)
1988
Current Themes in Paediatric Oncology
^cp?rt of a Symposium held in Bristol April 1987
tHe medical journal of the south west

				

